# *LAMA5* links extracellular matrix organization to a candidate WNT-associated endothelial signaling niche during human chondrogenesis

**DOI:** 10.1016/j.isci.2026.116687

**Published:** 2026-07-10

**Authors:** Alexander Schulz, Emily M. Brockmann, Steffen Uebe, Arif B. Ekici, Christian T. Thiel

**Affiliations:** 1 Institute of Human Genetics, Universitätsklinikum Erlangen, FAU Erlangen-Nürnberg, 91054 Erlangen, Germany

**Keywords:** LAMA5, chondrogenesis, extracellular matrix, Wnt signaling, growth plate, endothelial niche, idiopathic short stature

## Abstract

How extracellular matrix organization shapes signaling-associated transcription during skeletal development remains incompletely understood. Using CRISPR-Cas9-edited human urine-derived stem cells, we show that loss of the laminin gene *LAMA5* preserves canonical chondrogenic marker induction but impairs three-dimensional spheroid morphogenesis and disrupts pericellular matrix architecture. Bulk RNA sequencing and weighted gene co-expression network analysis identify a *LAMA5*-associated transcriptional module enriched for embryonic limb morphogenesis genes, with *WNT7A* and *FLI1* as inversely correlated hub genes. Projection onto a human fetal hindlimb atlas localizes *LAMA5*/*WNT7A* co-expression to the endothelial compartment, while *PITX1* and *FLI1* map to endothelial and chondrocyte populations, respectively, suggesting a spatially organized signaling axis partially conserved in mouse. Pharmacologic β-catenin stabilization partially restores selected module transcripts, and endothelial-conditioned medium selectively rescues *WNT7A* and *CDH1* in *LAMA5*-KO cultures. Together, these data link *LAMA5*-dependent matrix organization to a WNT-associated transcriptional program and identify endothelium as a candidate niche source.

## Highlights

• *LAMA5* loss disrupts spheroid architecture without blocking chondrogenic markers
• Transcriptomics defines a *LAMA5*-associated limb morphogenesis co-expression module
• Atlas projection localizes *LAMA5*/*WNT7A* to a candidate endothelial niche
• Endothelial-conditioned medium selectively induces *WNT7A* and *CDH1* in *LAMA5*-KO cells

## Graphical abstract

**Figure:**
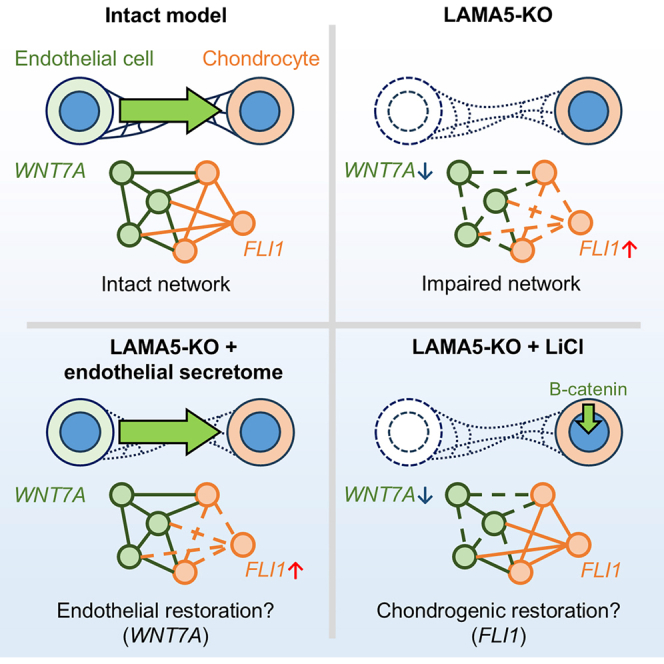

## Teaser

developmental genetics; stem cells research; developmental biology

## Introduction

Human height is strongly influenced by growth plate biology, and recent sequencing studies indicate that a substantial subset of individuals classified as having idiopathic short stature carry variants in genes expressed in cartilage, perichondrium, and adjacent vascular compartments. Variants in extracellular matrix (ECM) components such as *ACAN*, *COL2A1*, and other growth plate genes support the idea that quantitative disturbances of local skeletal tissue organization can affect longitudinal growth even in the absence of overt endocrine disease. Yet, the mechanisms by which ECM architecture is translated into signaling-associated transcriptional responses remain incompletely defined.^1^^,^^2^^,^^3^^,^^4^^,^^5^

Laminins are plausible mediators of this interface because they organize basement membrane-like pericellular environments and influence cell adhesion, polarity, and responsiveness to extracellular cues. *LAMA5* is expressed in developing cartilage and in the perichondrial and endothelial compartments that surround the growth plate, and biallelic pathogenic variants cause a severe skeletal disorder with growth plate disorganization. In addition, heterozygous *LAMA5* variants have been reported in cohorts with isolated short stature, suggesting that altered laminin dosage may also influence quantitative growth phenotypes. However, the downstream transcriptional consequences of *LAMA5* deficiency in human chondrogenic systems remain unclear.^6^^,^^7^^,^^8^^,^^9^^,^^10^

Canonical WNT signaling is particularly relevant in this context because it contributes to growth plate patterning, chondrocyte proliferation, and lineage progression. ECM organization may influence this pathway by shaping ligand presentation, receptor accessibility, and cell-cell boundary states. Notably, recent single-cell atlases of the developing limb have revealed that vascular endothelium and perichondrium express ECM and signaling components that may serve as a niche for adjacent chondrocytes, raising the question of whether matrix-producing cell populations contribute to the transcriptional environment of the growth plate beyond structural support alone.^9^^,^^11^^,^^12^^,^^13^

We therefore asked whether perturbation of *LAMA5* in a human chondrogenic model alters WNT-associated transcriptional programs and whether the resulting changes can be mapped onto spatially defined compartments in the developing limb. Using CRISPR-Cas9-engineered urine-derived stem cells (USCs), three-dimensional spheroid assays, bulk transcriptomics, co-expression analysis, single-cell atlas projection, and pharmacologic β-catenin stabilization, we define a *LAMA5*-dependent transcriptional module enriched for limb morphogenesis genes and localize it to a candidate endothelial signaling niche in the developing human growth plate.^14^^,^^15^

## Results

### *LAMA5* loss preserves induction of canonical differentiation markers

**Figure 1:**
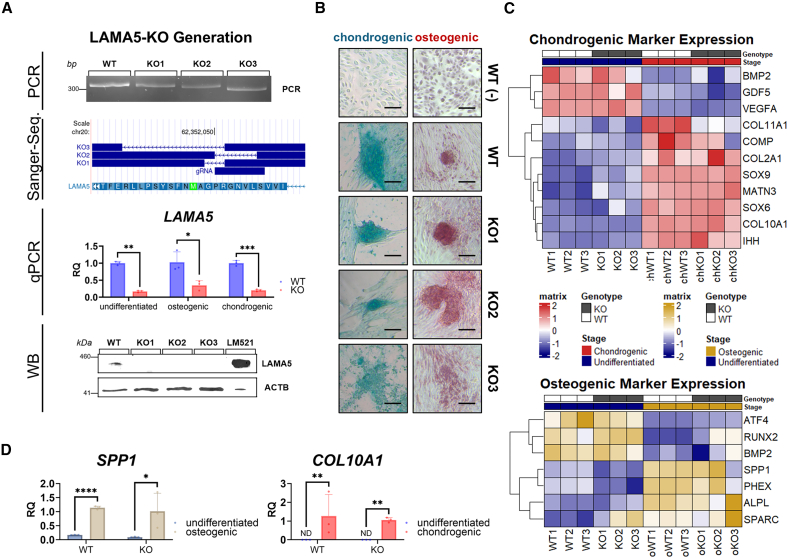
LAMA5 KO validation and preservation of canonical differentiation markers (A) Generation and validation of three independent *LAMA5 KO clones* in USCs. PCR: genotyping across the CRISPR target locus. Sanger-Seq.: sequencing traces aligned to the UCSC reference showing distinct frameshift-inducing deletions for each clone. qPCR: RT-qPCR confirming near-complete loss of *LAMA5 transcript* in undifferentiated, osteogenic, and chondrogenic conditions. WB: western blot of LAMA5 protein in all three KO clones, WT, and recombinant laminin-521 (LM-521) control. Unedited blots are provided in Figure S1B. (B) Alcian blue (chondrogenic) and Alizarin red (osteogenic) staining of WT and KO cultures with corresponding negative controls. Scale bars, 100 μm. WT and negative control images are shared with Schulz et al.^18^, which established the baseline differentiation characterization of the parental USC line used for CRISPR editing in this study. (C) Heatmaps of canonical osteogenic and chondrogenic marker gene sets from bulk RNA-seq comparing undifferentiated and differentiated WT and KO cultures. Expression patterns are broadly conserved between genotypes; *COL11A1* shows reduced expression in KO chondrogenic cultures. (D) RT-qPCR validation of terminal differentiation markers *SPP1* (osteogenic) and *COL10A1* (chondrogenic). *SPP1* was induced in both genotypes (WT, *p* = 2 × 10^−6^; KO, *p* = 0.026), and *COL10A1* was induced in both genotypes (WT, *p* = 0.0019; KO, *p* = 0.0018; unpaired Welch’s two-sample *t* test on ΔΔCt values; *n* = 3 biological replicates, i.e., the three independent CRISPR-edited KO clones). Data are mean ± SD. ND, not detected. See also Figure S1.

To examine how *LAMA5* influences human chondrogenesis, we generated three independent *LAMA5* knockout (KO) USC clones by CRISPR-Cas9 editing. Targeted PCR and Sanger sequencing confirmed distinct frameshift-inducing deletions at the *LAMA5* locus, and reverse-transcription quantitative polymerase chain reaction (RT-qPCR) together with western blotting showed near-complete depletion of *LAMA5* transcript and protein across undifferentiated, osteogenic, and chondrogenic conditions (Figures 1A and S1B). Single-cell clonal expansion was supported by transient laminin-521 coating during derivation (Figure S1A). Under two-dimensional differentiation conditions, wild-type (WT) and KO cultures showed comparable Alcian blue and Alizarin red staining patterns, including negative controls (Figure 1B). The cell condensation and detachment from the culture surface observed at the onset of chondrogenic induction reflects the expected aggregation step of mesenchymal-to-chondrogenic differentiation and is a prerequisite for cartilage matrix deposition, rather than an artifact of plate adhesion.^16^^,^^17^ Bulk RNA sequencing (RNA-seq) heatmaps of canonical osteogenic and chondrogenic marker sets supported broadly similar expression patterns in both genotypes, although *COL11A1* showed reduced expression in KO chondrogenic cultures (Figures 1C and S1C). RT-qPCR analysis of *SPP1* and *COL10A1* further confirmed that *LAMA5* loss did not abolish acquisition of terminal differentiation marker expression (Figure 1D). These findings argue against a primary block in lineage-associated transcription under the assay conditions used here.

Beyond marker expression, *LAMA5* KO2 cells showed a reduced proliferative capacity compared with WT cells in standard 2D expansion culture, and pre-coating the culture surface with recombinant laminin-521 did not restore proliferation toward WT levels (Figure S1D). This indicates that the *LAMA5*-dependent phenotype is not corrected by passive matrix presentation alone and may also partially contribute to the smaller size of KO chondrogenic spheroids observed in subsequent three-dimensional assays.

### *LAMA5* deficiency impairs spheroid morphogenesis and disrupts adhesion-associated programs

**Figure 2:**
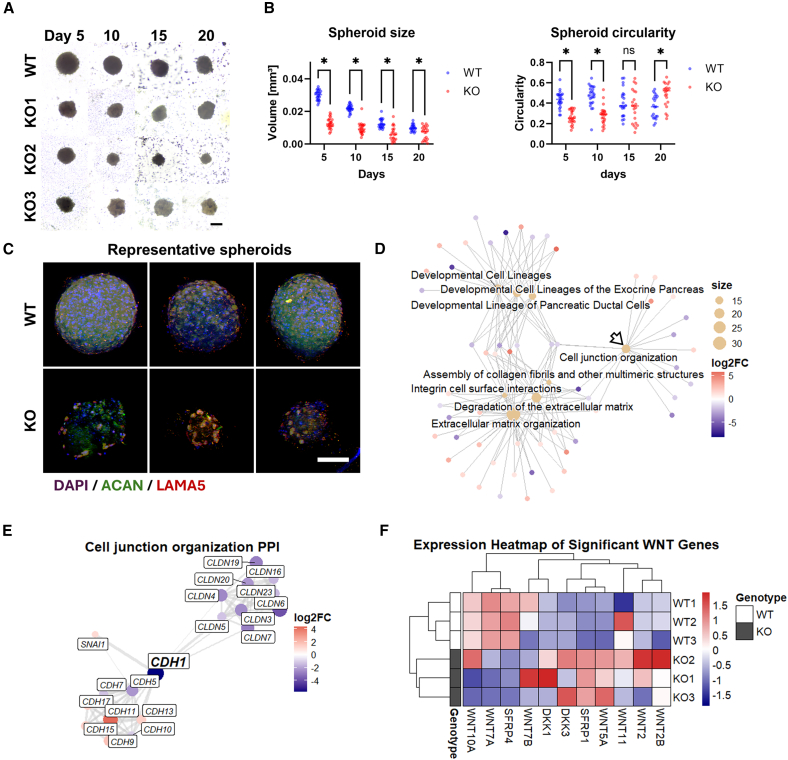
LAMA5 deficiency impairs chondrogenic spheroid morphogenesis and reveals altered adhesion and WNT-associated transcriptional programs (A) Representative brightfield images of chondrogenic spheroids from WT and KO clones 1–3 at days 5, 10, 15, and 20 of differentiation. Scale bars, 200 μm. (B) Quantification of spheroid volume and size across all replicates and time points. KO spheroids were smaller across the time course (representative exact *p* values: day 5, *p* < 0.0001; day 20, *p* = 0.0044) and less circular during early morphogenesis (day 5–10, *p* < 1 × 10^−6^) with persistent disruption at day 20 (*p* = 0.001) (unpaired Welch’s *t* test with Holm-Šidák correction; *n* = 21–24 spheroids per group). (C) Immunofluorescence merge images of representative day-20 spheroid morphologies from WT (three examples) and KO (three examples). WT spheroids display compact architecture with continuous aggrecan (ACAN, green) distribution and laminin-α5 (LAMA5, red) signal concentrated at the periphery and in pericellular boundaries. KO spheroids are disaggregated with patchy, irregular ACAN deposition and absent or fragmentary LAMA5 signal. DAPI (blue). Scale bars, 100 μm. (D) Reactome pathway enrichment network of differentially expressed genes in KO versus WT chondrogenic cultures. The cell junction organization term is highlighted. (E) STRING protein-protein interaction network of differentially expressed genes within the cell junction organization pathway. Node colors indicate log_2_ fold change; CDH1 and multiple claudin family members are downregulated in KO cultures. (F) Heatmap of canonical WNT pathway genes during chondrogenic differentiation in WT and KO cultures. Statistically significant genes (adjusted *p* < 0.05) are shown; the overall pattern reflects dysregulation rather than uniform downregulation. An extended KEGG pathway overlay is provided in Figure S2. See also Figures S2 and S3.

When cells were cultured as three-dimensional chondrogenic spheroids, KO cultures reproducibly formed smaller and less regular aggregates than WT controls across all three clones and time points (Figure 2A). Quantification showed reduced spheroid volume and lower circularity during early morphogenesis, with persistent architectural disruption at day 20 (Figure 2B). Immunofluorescence of day-20 spheroids stained for aggrecan (*ACAN*) and laminin-α5 (*LAMA5*) revealed striking structural differences between genotypes (Figure 2C). WT spheroids displayed compact architecture with continuous ACAN distribution throughout the spheroid body and LAMA5 signal concentrated at the periphery and in pericellular boundaries. In contrast, KO spheroids were markedly smaller, disaggregated, and showed patchy, irregular ACAN deposition. LAMA5 signal was markedly reduced and discontinuous in KO cultures, consistent with disruption of the organized pericellular matrix boundary observed in WT controls. Reduced spheroid size in KO cultures may in part reflect the reduced 2D proliferative capacity of KO cells (Figure S1D), although the qualitative differences in pericellular matrix organization (continuous vs. discontinuous LAMA5 deposition, patchy ACAN distribution) and circularity (Figure 2B) cannot be explained by lower cell number alone.

To define the molecular basis of this phenotype, we performed bulk RNA sequencing on differentiated WT and KO cultures. Reactome pathway enrichment of differentially expressed genes highlighted changes in ECM organization, cell junction organization, and related adhesion-associated processes (Figure 2D). Protein-protein interaction analysis of the cell junction organization term showed a downregulated cluster centered on *CDH1* and multiple claudin family members, supporting disruption of an intercellular boundary-associated transcriptional state (Figure 2E). Inspection of canonical WNT pathway genes revealed a more complex pattern than uniform suppression: WNT target-associated transcripts were dysregulated rather than universally downregulated in KO cultures, suggesting perturbation of the transcriptional configuration through which WNT-associated signals are interpreted (Figure 2F). An extended Kyoto encyclopedia of genes and genomes (KEGG) pathway overlay further supported broad pathway disturbance rather than a strictly one-directional response (Figure S2). Examination of canonical β-catenin/TCF target genes at the transcript level supported this interpretation: *AXIN2*, *LEF1*, *TCF7*, *NKD1*, and *SP5* were not significantly reduced in KO cultures, and *CCND1* showed a modest but significant increase (Figure S3A). This pattern indicates that *LAMA5* loss does not produce a canonical β-catenin/TCF suppression signature, consistent with a perturbation acting upstream of intracellular β-catenin activity, at the level of WNT ligand availability or ECM-associated signaling niche organization.

### Co-expression analysis defines a *LAMA5*-associated limb morphogenesis module

**Figure 3:**
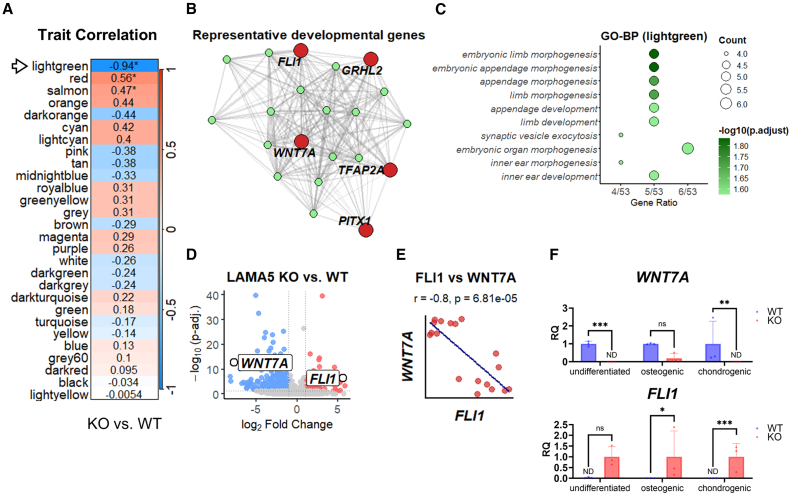
Co-expression analysis defines a LAMA5-associated limb morphogenesis module (A) WGCNA module-trait correlation heatmap across all cell types and conditions. The light green module shows the strongest negative association with the KO genotype (Pearson r = −0.94, *p* = 7.38 × 10^−9^). Scale-free topology assessment supporting power selection is shown in Figure S4A. (B) Network visualization of representative light green-module genes with their neighbors. Highlighted genes contribute to the embryonic limb morphogenesis GO term shown in (C), including *WNT7A*. An extended module network is shown in Figure S4B. (C) GO enrichment analysis for the light green module. Embryonic limb morphogenesis is the top-ranked term (5/53 module genes). (D) Volcano plot of light green-module genes across all cell types. WNT7A and FLI1 are labeled as the most prominent hub genes with opposing directions of regulation. (E) Pairwise correlation of *WNT7A* and *FLI1* across all samples (Pearson r = −0.8, *p* = 6.8 × 10^−5^; *n* = 18), confirming inverse co-expression within the module. A broader hub gene correlation analysis is provided in Figure S4C. (F) RT-qPCR validation under chondrogenic conditions confirms reduced *WNT7A* (*p* = 0.008) and increased *FLI1* (*p* = 8.8 × 10^−4^) in KO cultures (unpaired Welch’s two-sample *t* test on ΔΔCt values; *n* = 3 biological replicates, i.e., the three independent CRISPR-edited KO clones). Data are mean ± SD. See also Figure S4.

Because the transcriptomic changes appeared coordinated, we applied weighted gene co-expression network analysis (WGCNA) to the full RNA-seq dataset spanning undifferentiated, osteogenic, and chondrogenic conditions (soft-thresholding power 9; Figure S4A). Among the resulting modules, the light green module showed the strongest negative association with the KO genotype (Pearson r = −0.94, *p* = 7.38 × 10^−9^; Figure 3A). Network visualization of representative module genes and their neighbors revealed dense connectivity among development-associated transcripts (Figures 3B and S4B). Gene ontology enrichment linked this module to embryonic limb morphogenesis as the top-ranked term (five of 53 module genes; Figure 3C), suggesting that *LAMA5* loss perturbs a coordinated skeletal patterning program rather than only generic stress responses.

Within this module, *WNT7A* and *FLI1* emerged as prominent hub genes with opposing directions of regulation in KO cultures (Figure 3D). *WNT7A* showed high module membership (MM = 0.91), consistent with its role as a central hub gene, whereas *FLI1* (MM = 0.72) was upregulated. Their expression values were strongly inversely correlated across all samples (Pearson r = −0.8, *p* = 6.8 × 10^−5^; Figure 3E), and pairwise correlation analysis of the top 15 hub genes supported broader co-regulatory structure within the module (Figure S4C). *PITX1*, *TFAP2A*, and *GRHL2* were also members of the light green module and contributed to the limb morphogenesis enrichment. RT-qPCR validation confirmed reduced *WNT7A* and increased *FLI1* under chondrogenic conditions (Figure 3F).

These data define a reproducible *LAMA5*-associated transcriptional module centered on genes with known developmental relevance, while remaining agnostic about direct regulatory directionality among individual members.

### Atlas projection places *LAMA5*/*WNT7A* and *PITX1*/*FLI1* in spatially resolved growth plate compartments

**Figure 4:**
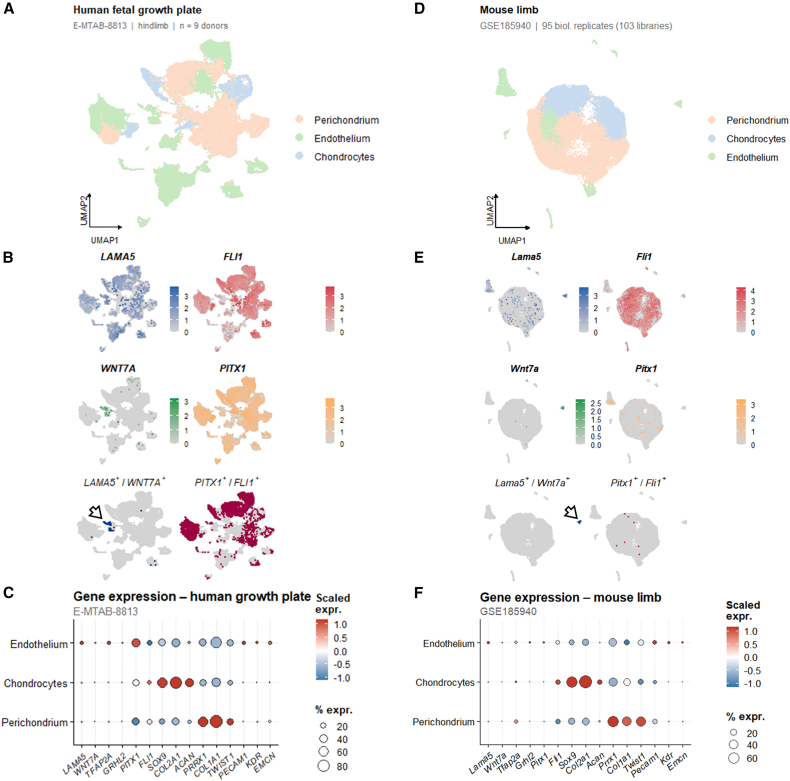
Developmental atlas context of the LAMA5-associated transcriptional module (A) Uniform Manifold Approximation and Projection (UMAP) embedding of the human fetal hindlimb single-cell atlas (E-MTAB-8813; *n* = 9 donors, 5.1–9.3 post-conception weeks) with cell-type annotation into chondrocytes, perichondrium, and endothelium. (B) Six-part FeaturePlot of module genes in the human atlas. Top row: *LAMA5*, *FLI1*. Middle row: *WNT7A*, *PITX1*. Bottom row: co-expression overlays for *LAMA5*^+^/*WNT7A*^+^ and *PITX1*^+^/*FLI1*^+^ cells. *LAMA5* is detected in endothelium and to a lesser extent in perichondrium and chondrocytes; *LAMA5*/*WNT7A* co-expression localizes primarily to the endothelial compartment. *PITX1* and *FLI1* show broad distribution, with *PITX1* enriched in endothelium and present in chondrocytes. (C) Dot plot of module genes and cell-type markers across human growth plate compartments. *PITX1* is enriched primarily in endothelium with lower expression in chondrocytes; *FLI1* shows strongest expression in chondrocytes. (D) UMAP embedding of the mouse forelimb atlas (GSE185940) with equivalent cell-type annotation. (E) Six-part FeaturePlot of orthologous module genes in mouse. *Lama5* and *Wnt7a* show a comparable perichondrial pattern; *Pitx1* expression is lower overall, and *Pitx1*/*Fli1* co-expression is markedly reduced compared to the human atlas. (F) Dot plot of module genes and cell-type markers across mouse forelimb compartments.

To place the module in developmental context, we projected *LAMA5* and selected module genes onto a human fetal hindlimb single-cell atlas comprising 9 donors across 5.1–9.3 post-conception weeks (Figure 4A). This analysis resolved expected tissue compartments, including chondrocytes, perichondrium, and endothelium. *LAMA5* was detected in the endothelial compartment and, to a lesser extent, in perichondrium and chondrocytes. Notably, *LAMA5*/*WNT7A* co-expression localized primarily to endothelial cells, identifying this population as a candidate source of the ECM-associated signaling program disrupted in our *in vitro* model (Figure 4B). *PITX1* showed enrichment primarily in endothelium with lower expression in chondrocytes, whereas *FLI1* was most strongly represented in the chondrocyte population (Figures 4B and 4C). The endothelial co-localization of *LAMA5*, *WNT7A*, and *PITX1* may reflect a spatial continuum linking the ECM-producing compartment to downstream chondrocyte transcriptional states, although direct intercellular signaling cannot be inferred from expression data alone.^14^

Projection onto a mouse forelimb atlas revealed partial conservation and partial divergence (Figures 4D–4F).

*Lama5* and *Wnt7a* showed a comparable pattern in murine perichondrial and endothelial compartments. However, *Pitx1* expression was markedly lower overall, and *Pitx1*/*Fli1* co-expression was substantially reduced compared to the human atlas. These species differences suggest that while the upstream ECM-associated program may be conserved, its downstream transcriptional interpretation in chondrocytes is not fully mirrored across species.^15^

### Human genetic context supports developmental relevance of the module

**Figure 5:**
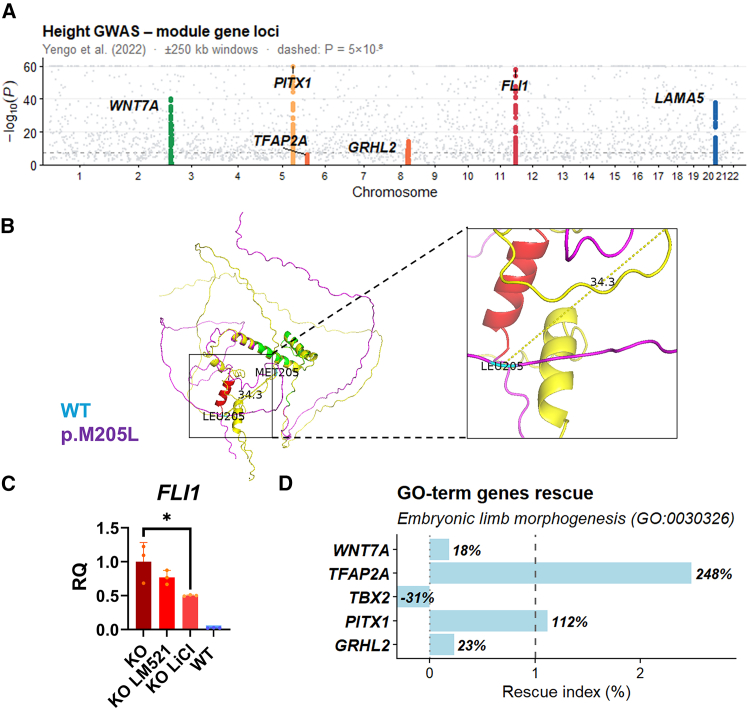
Human genetic context and partial pharmacologic rescue of the LAMA5-associated light green module (A) Manhattan plot of height GWAS summary statistics (Yengo et al., 2022). Module gene loci are highlighted within ±250 kb windows. *PITX1* and *FLI1* show the strongest associations; *LAMA5* and *WNT7A* show moderate signals; *TFAP2A* and *GRHL2* show modest enrichment. Dashed line indicates genome-wide significance (*p* = 5 × 10^−8^). (B) Structural modeling of wild-type PITX1 and the p.M205L variant identified in an individual with isolated short stature (Table S1). The substitution maps outside the canonical homeodomain, near the C-terminal OAR region. Insets show zoom views of misaligned domains. Sanger confirmation is provided in Figure S5C. (C) RT-qPCR analysis of *FLI1* expression in the KO2 clone after LiCl treatment. LiCl increased *FLI1* toward WT levels (*p* = 0.031; unpaired Welch’s two-sample *t* test on ΔΔCt values; *n* = 3), whereas exogenous laminin-521 coating did not restore expression. Data are mean ± SD. (D) Rescue index after LiCl treatment of KO2 cells for the genes of the embryonic limb morphogenesis GO term (GO:0030326) enriched in the LAMA5-associated light green module. Rescue index = (KO2+LiCl − KO2)/(WT − KO2), calculated from batch-corrected variance-stabilized expression. 100% (dashed line) indicates restoration to WT level; 0% (dotted line) indicates no change relative to untreated KO2; negative values indicate further divergence from WT. Underlying transcripts per million (TPM) distributions for the ELM gene set are shown in Figure S5A; the analogous rescue-index analysis for the top 15 hub genes of the same module is shown in Figure S5B. See also Figures S3 and S5.

To place the *LAMA5*-associated module in a developmental and human-genetic context, we asked whether its constituent genes intersect with human growth phenotypes. Gene ontology (GO) biological process enrichment of the light green module highlighted *embryonic limb morphogenesis* (GO:0030326) as the most strongly represented developmental term, comprising *WNT7A*, *PITX1*, *TFAP2A*, *GRHL2*, and *TBX2*—a set we hereafter refer to as the embryonic-limb-morphogenesis (ELM) gene set and use as a focused, biologically motivated readout of module-level rescue. As orthogonal human-genetic context, we examined whether these and other module genes overlap loci implicated by genome-wide association studies (GWAS) for adult height. Multiple genes from the experimentally defined module mapped to height-associated loci, with *PITX1* and *FLI1* showing the strongest associations, *LAMA5* and *WNT7A* showing moderate signals, and *TFAP2A* and *GRHL2* showing modest enrichment (Figure 5A). In addition, we identified a heterozygous *PITX1* p.M205L variant (NM_002653.3, c.A613C) in a 12-year-old female with isolated short stature (height −3.3 standard deviation score (SDS); Table S1). Structural modeling placed this substitution outside the canonical homeodomain, near the C-terminal otp, aristaless, and rax (OAR) region, in a domain that showed local structural rearrangement relative to the wild-type model (Figures 5B and S5C). This single case does not establish causality for the network, but it is consistent with the broader idea that genes within the identified module intersect human growth phenotypes.^18^^,^^19^

### Pharmacologic β-catenin stabilization partially restores ELM-module gene expression

Given the human-genetic overlap of the *LAMA5*-associated module with height-related loci, we asked whether the *LAMA5*-dependent transcriptional state can be pharmacologically modulated. Because KO cultures displayed a perturbed WNT-associated signature, we tested whether pathway activation downstream of membrane-proximal events could modify the transcriptional phenotype. Lithium chloride (LiCl), a GSK3β inhibitor, significantly increased *FLI1* expression in KO cells toward WT levels, whereas coating with recombinant laminin-521 did not restore *FLI1* expression under the same assay conditions (Figure 5C). This differential response suggests that downstream β-catenin stabilization can partially bypass the transcriptional consequences of *LAMA5* loss, whereas exogenous matrix coating alone is insufficient.^20^^,^^21^

Consistent with effective pathway activation, LiCl significantly induced four of six canonical β-catenin/TCF target genes (*AXIN2*, *TCF7*, *NKD1*, and *CCND1*) in KO cultures (Figure S3B), confirming that the partial and gene-specific module rescue described below does not reflect failure of pharmacologic β-catenin stabilization.

Rescue-index analysis of LiCl-treated cultures was performed across the ELM gene set defined above (*WNT7A*, *PITX1*, *TFAP2A*, *GRHL2*, and *TBX2*) as a focused module-level readout. Positive rescue values were observed for *WNT7A*, *PITX1*, *TFAP2A*, and *GRHL2* (Figure 5D), with the underlying transcripts per million (TPM) distributions shown in Figure S5A. *TBX2*, which was the sole gene upregulated in KO cultures within the module, showed a negative rescue index consistent with further stimulation by LiCl, potentially reflecting a compensatory mechanism. Extending the analysis to the top 15 hub genes of the light green module showed a heterogeneous pattern of partial rescue, no rescue, or further deviation from WT (Figure S5B), with *WNT7A* among the strongest positive responders. Together, these results indicate that selected components of the *LAMA5*-associated module remain pharmacologically responsive to β-catenin stabilization, while the incomplete and gene-specific response is consistent with pathway cross-talk and with the broad pharmacologic effects of GSK3β inhibition.

### Endothelial-conditioned medium partially normalizes ELM-module and junction-associated transcripts during *LAMA5*-KO chondrogenesis

**Figure 6:**
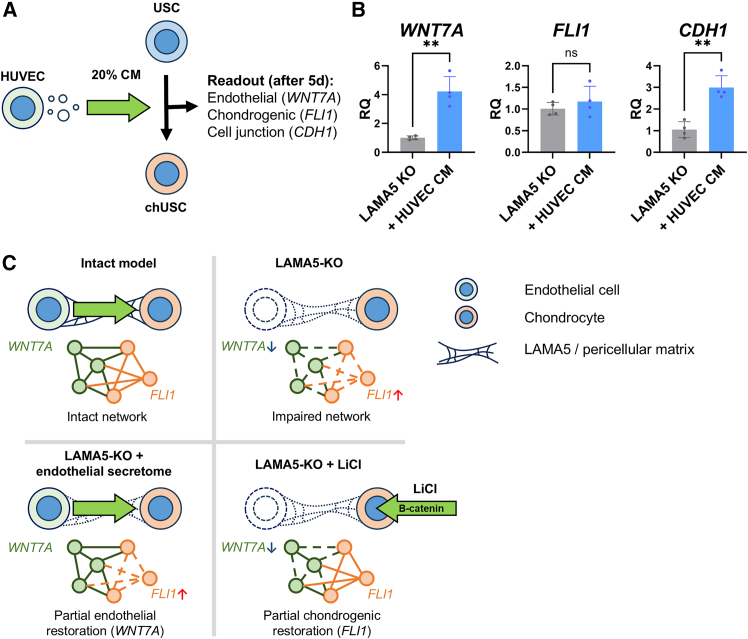
Endothelial conditioned medium partially restores ELM-module and adhesion-associated transcripts in LAMA5-KO chondrogenic cultures (A) Experimental schematic. Human umbilical vein endothelial cells (HUVECs; passage 0, freshly isolated from umbilical cords; cultured in PromoCell Endothelial Cell Growth Medium Ready-to-use) were used as a source of endothelial-conditioned medium (CM). LAMA5-KO2 USCs were maintained in 2D chondrogenic induction medium supplemented with 20% HUVEC-CM, with medium refresh every 48 h. Two 48-h CM-treatment cycles were applied prior to RT-qPCR readout at day 5 of chondrogenic differentiation (final CM refresh 48 h before harvest). Three orthogonal LAMA5-affected programs were probed: the ELM-module representative *WNT7A* (light green-module hub, MM = 0.91), the chondrogenically inert opposing hub *FLI1* (light green module, not part of the ELM gene set), and the cell-junction-associated transcript *CDH1* (Figure 2E cluster, module-independent). (B) RT-qPCR of *WNT7A*, *FLI1*, and *CDH1* in KO2 cultures with or without 20% HUVEC-CM. *WNT7A* was strongly induced by HUVEC-CM (mean relative quantification (RQ) ≈ 4.2 vs. 1.0; *p* = 1.9 × 10^−4^, ∗∗), *CDH1* was induced (mean RQ ≈ 2.5 vs. 1.0; *p* = 4.3 × 10^−3^, ∗∗), and *FLI1* was not significantly affected (mean RQ ≈ 1.2 vs. 1.0; *p* = 0.47, ns). RQ values are relative to the untreated KO2 group set to 1. *n* = 4 independent differentiation runs per condition; unpaired Welch’s two-sample *t* test on ΔΔCt values; outlier removal by 1.5 × IQR per sample × target; normalization to RPLP0. Data are mean ± SD. ∗∗, *p* < 0.01; ns, not significant. (C) Working model of *LAMA5*-dependent matrix-signaling crosstalk during chondrogenesis. Intact model (top left): an endothelial cell expresses *LAMA5* and *WNT7A* (Figure 4B) and deposits *LAMA5* into a continuous pericellular matrix that supports *WNT7A* signaling toward the adjacent chondrocyte, sustaining an intact ELM network (green nodes, solid edges) and limiting *FLI1*. *LAMA5*-KO (top right): loss of *LAMA5* disrupts the pericellular matrix (fragmented mesh, dashed) and the endothelial-derived *WNT7A* input is no longer transmitted to the chondrogenic compartment (arrow down); the chondrocyte loses the ELM-network state while *FLI1* is derepressed (red up). *LAMA5*-KO + endothelial secretome (bottom left, this study): exposure of *LAMA5*-KO chondrogenic cultures to HUVEC-conditioned medium partially restores the endothelial-input component, with chondrocyte *WNT7A* and *CDH1* transcript levels increased without changing *FLI1*, consistent with the candidate endothelial niche identified by atlas projection (Figure 4). *LAMA5*-KO + LiCl (bottom right, Figures 5C and 5D): pharmacologic β-catenin stabilization bypasses the disrupted matrix-ligand interface and acts on the downstream chondrocyte transcriptional program (restoration of *FLI1* and partial restoration of ELM transcripts), without engaging the upstream ECM defect.

Atlas projection identified vascular endothelium as a candidate source of the *LAMA5*/*WNT7A*-associated signaling environment (Figure 4B), but did not directly test whether endothelial-derived soluble factors influence the *LAMA5*-dependent transcriptional state of chondrogenic receiver cells. To probe this candidate niche functionally, we cultured *LAMA5*-KO2 USCs in 2D chondrogenic induction medium supplemented with 20% human umbilical vein endothelial cell-conditioned medium (HUVEC-CM) for two 48-h treatment cycles and harvested at day 5 of differentiation (Figure 6A).

We selected three orthogonal *LAMA5*-affected readouts. *WNT7A* served as the central ELM-module hub, with high module membership (MM = 0.91), downregulation in KO, and a prominent position in the WGCNA network (Figure 3F). *FLI1* represented the opposing light green hub gene: it is upregulated in KO but is not a member of the ELM gene set (GO:0030326) and is annotated as chondrogenically inert in the human fetal limb atlas. *CDH1* was included as a representative of the adhesion-associated cluster disrupted in KO chondrogenic cultures (Figure 2E), serving as a molecular surrogate for the early mesenchymal-condensation step that is impaired in *LAMA5*-KO spheroids (Figures 2A–2C).

HUVEC-CM significantly induced *WNT7A* (mean RQ ≈ 4.2 vs. 1.0; *p* = 1.9 × 10^−4^) and *CDH1* (mean RQ ≈ 2.5 vs. 1.0; *p* = 4.3 × 10^−3^) in KO2 cultures, whereas *FLI1* was not significantly affected (mean RQ ≈ 1.2 vs. 1.0; *p* = 0.47; *n* = 4 independent differentiation runs per condition; Figure 6B). This pattern is selective rather than generic: the two transcripts that are downregulated in KO and that correspond to compartments expected to receive endothelial input (the ELM hub *WNT7A* and the junction-associated *CDH1*) were partially restored, while the chondrocyte-intrinsic, atlas-defined *FLI1* axis, which is not expected to be addressable by endothelial-derived soluble factors but does respond to direct intracellular β-catenin stabilization with LiCl (Figure 5C), remained unchanged. The response is therefore consistent with a model in which soluble endothelial factors contribute to the *LAMA5*-affected candidate niche at the level of WNT-associated ligand availability and early junction-organization programs during chondrogenic induction, rather than acting as a pan-stimulus on all KO-deregulated transcripts. This provides initial functional support for the candidate endothelial signaling niche inferred from atlas projection (Figure 4). The experiment was performed as a within-KO comparison without an in-experiment WT arm or basal-medium mock control and is therefore interpreted as hypothesis-supporting (see discussion for full caveats). A working model that integrates the experimental, atlas-derived and HUVEC-CM observations is shown in Figure 6C.

## Discussion

Our data support a model in which *LAMA5* primarily contributes to organization of a signaling-competent extracellular environment during human chondrogenesis rather than to the binary acquisition of lineage-marker expression. Across three independent KO clones, the strongest phenotypes were architectural: reduced spheroid size, altered circularity, and loss of continuous pericellular aggrecan and laminin-α5 deposition. These structural changes were accompanied by altered adhesion-associated pathways centered on *CDH1* and claudin family members, together with dysregulation of canonical WNT-associated genes. The combination of intact marker induction and impaired morphogenesis links matrix organization to transcriptional state without requiring the conclusion that *LAMA5* directly controls lineage commitment in this system.^9^^,^^22^^,^^23^

A central finding of this study is the identification of a *LAMA5*-associated co-expression module enriched for embryonic limb morphogenesis genes, with *WNT7A* and *FLI1* as inversely correlated hub genes. Notably, canonical β-catenin/TCF target genes were not coordinately suppressed in KO cultures at the transcript level (Figure S3A), arguing against a simple loss of canonical WNT/β-catenin output as the primary defect. Together with the reduced expression of *WNT7A* itself and the robust induction of multiple canonical targets upon pharmacologic β-catenin stabilization (Figure S3B), these observations are most consistent with a perturbation acting upstream of intracellular β-catenin activity, at the level of ligand availability and ECM-associated niche organization, rather than at the level of β-catenin stability or TCF-driven transcription. This view is compatible with the partial and selective effects of LiCl rescue: bypassing the upstream defect by intracellular β-catenin stabilization restores selected module genes (*WNT7A*, *PITX1*, *TFAP2A*, and *GRHL2*) but cannot reconstitute the full *LAMA5*-dependent transcriptional program, consistent with pathway cross-talk and with the pleiotropic effects of GSK3β inhibition.^23^ The negative rescue index of *TBX2*, which was upregulated in KO cultures and further stimulated by LiCl, may reflect a compensatory transcriptional response that operates independently of the *LAMA5*-dependent ECM circuit.

Projection onto human and mouse developmental atlases provided spatial context for the module. In the human fetal hindlimb, *LAMA5* and *WNT7A* co-expression localized primarily to the endothelial compartment, identifying vascular endothelium as a candidate source of the ECM-associated signaling program disrupted in our *in vitro* model. *PITX1* showed enrichment in endothelium with lower expression in chondrocytes, whereas *FLI1* was most strongly represented in the chondrocyte population. This spatial arrangement raises the possibility of a signaling axis in which endothelial *LAMA5* and *WNT7A* contribute to an ECM environment that shapes transcriptional states in adjacent chondrocytes.^24^ The partial overlap of *PITX1* between endothelial and chondrocyte populations is consistent with a spatial continuum rather than a strict boundary between the module’s upstream and downstream components. However, our study does not directly test intercellular communication, and expression-based co-localization cannot establish functional signaling relationships. Consistent with this candidate niche model, exposure of *LAMA5*-KO chondrogenic cultures to HUVEC-conditioned medium selectively induced the ELM-module hub *WNT7A* and the junction-associated *CDH1*, the two transcripts that are downregulated in KO and that correspond to compartments expected to receive endothelial input, while leaving the chondrocyte-intrinsic *FLI1* axis unchanged. This pattern is consistent with the prediction that endothelial-secretome input acts on the niche-associated *WNT7A*/*CDH1* arm rather than on the chondrocyte-autonomous, β-catenin-engaging *FLI1* arm, which is instead modulated by direct intracellular β-catenin stabilization with LiCl (Figure 5C). This selective transcriptional response provides initial functional support for an endothelial input on the *LAMA5*-affected program, although it does not reconstitute the ECM-associated component of the candidate niche and does not establish a direct intercellular signaling event.

The mouse forelimb atlas comparison revealed partial conservation and partial divergence. *Lama5* and *Wnt7a* showed comparable localization in murine endothelial and perichondrial compartments, suggesting that the upstream ECM-associated program may be conserved. However, *Pitx1* expression was substantially lower and *Pitx1*/*Fli1* co-expression was markedly reduced in the mouse, implying that the downstream transcriptional interpretation of this program is not fully mirrored across species. This observation aligns with prior reports of species-specific differences in growth plate gene regulation and underscores that conclusions drawn from one model system should be extrapolated cautiously.

### Limitations of the study

Several important limitations should be considered. USC cultures do not fully recapitulate the native growth plate, and our study does not define the exact physiologic progenitor-to-chondrocyte transition occurring *in vivo*. The atlas projections provide developmental context for the observed transcriptomic changes rather than proof of a resolved niche-receiver circuit. Furthermore, the endothelial identity of the *LAMA5*/*WNT7A*-co-expressing cells is based on marker-driven annotation within the atlas and would require validation *in situ*. We also note that LiCl affects multiple signaling pathways beyond canonical WNT, so the rescue experiments should be interpreted as evidence for partial pharmacologic responsiveness rather than definitive pathway restoration. Similarly, the HUVEC-CM rescue (Figure 6) provides functional but partial support for the endothelial-input hypothesis. HUVECs are umbilical-vein endothelium and differ in vascular origin from the microvascular endothelium identified in the human fetal limb atlas^25^; conditioned medium captures only soluble factors and does not reconstitute the ECM-bound component of the candidate niche; and WNT-family ligands are typically not efficiently captured in conditioned medium owing to their lipidation and heparan-sulfate-proteoglycan dependence,^26^ so the observed *WNT7A* response is most plausibly mediated by endothelial-derived non-WNT factors acting on receiver-cell transcription rather than by direct *WNT7A* protein transfer. The experiment was furthermore performed as a within-KO comparison without an in-experiment WT arm and without a basal-medium mock control, so effects are best interpreted as hypothesis-supporting rather than mechanistically definitive.

Consistent with this view, recombinant laminin-521 coating did not restore the reduced proliferative capacity of KO cells in standard 2D culture (Figure S1D), suggesting that the *LAMA5*-dependent phenotype is not corrected by passive matrix presentation alone and reflects more than simple availability of laminin α5 at the cell surface. Reduced 2D proliferation in KO cultures may also partially contribute to the smaller spheroid size observed in three-dimensional chondrogenic assays. However, the qualitative differences in pericellular matrix organization, the disrupted spheroid circularity, and the partial transcriptional rescue by LiCl together argue against a model in which reduced proliferation alone accounts for the observed phenotypes.

The human genetic observations provide supportive but not definitive context. Overlap between the experimentally defined module and height-associated GWAS loci suggests that these genes intersect pathways relevant to normal variation in stature. The *PITX1* p.M205L case offers a clinically relevant point of convergence with prior literature linking *PITX1* variation to lower-limb developmental phenotypes, but a single case cannot establish a generalized mechanism for idiopathic short stature, a heterogeneous condition.^18^^,^^19^

Building on the HUVEC-CM result (Figure 6), future work in more physiologic systems (including direct endothelial-chondrogenic co-culture, organoid, *ex vivo* growth plate, or *in vivo* models with cell-type-specific perturbation of candidate downstream genes) will be required to determine whether the spatially segregated programs inferred here correspond to functional signaling relationships at single-cell resolution. Testing whether endothelial-specific *LAMA5* depletion recapitulates the transcriptional phenotype observed in our stem cell model would be a particularly informative next step. Overall, the present study positions *LAMA5*-dependent matrix organization as a candidate regulator of WNT-associated transcriptional states during human chondrogenesis and provides a framework for more direct mechanistic testing in growth plate-relevant models.^22^^,^^23^

## Resource availability

### Lead contact

Further information and requests for resources should be directed to and will be fulfilled by the lead contact, Christian T. Thiel (christian.thiel@uk-erlangen.de).

### Materials availability

This study did not generate unique reagents beyond the CRISPR-edited cell clones described in the manuscript. Availability of cell material is subject to institutional approvals and material transfer regulations. Detailed reagents, assays, antibodies, software, and database identifiers are listed in the key resources table.

### Data and code availability

RNA-seq raw data are available at ArrayExpress: E-MTAB-16566. Original source data for western blots, brightfield spheroid imaging, immunofluorescence, and RT-qPCR have been deposited at Mendeley Data: https://doi.org/10.17632/x92s3b23cz.1. All original code, including custom FIJI macros and R analysis scripts used for the reported results, is available at GitHub: https://github.com/alexschulzcell/LAMA5-paper and has been deposited at Zenodo: https://doi.org/10.5281/zenodo.20697901. The DOI is also listed in the key resources table and is publicly available as of the date of publication. This study does not report a new standalone algorithm. Supplemental information contains extended analytical details, supplemental figure legends, and supplemental tables.

## Acknowledgments

We thank all patients and their families for participating in this study. We acknowledge the FAU FACS Core Unit (NFZ) for expert assistance with cell sorting and technical advice. We thank Evelyn Galsterer for excellent technical assistance. We are grateful to Prof. Iwona Cicha and Yi-Yu Robin Dai (Cardiovascular Nanomedicine Unit, Section of Experimental Oncology and Nanomedicine, Department of Otorhinolaryngology, Head and Neck Surgery, University Hospital Erlangen) for kindly providing HUVEC-conditioned medium and for sharing technical details on HUVEC isolation and culture. The use of human umbilical cord material for HUVEC isolation was approved by the Institutional Ethical Committee on Human Research at the University Hospital Erlangen (ethical review number 21-331-B, 06.10.2021). This work was supported by the 10.13039/501100001659Deutsche Forschungsgemeinschaft (DFG, German Research Foundation) grant TH 896/7-1 (C.T.T.). An earlier version of this work was posted on bioRxiv: https://doi.org/10.1101/2025.11.19.689218.

## Author contributions

Conceptualization, C.T.T. and A.S.; methodology, A.S., E.M.B., S.U., and A.B.E.; software, A.S.; validation, A.S.; formal analysis, A.S. and E.M.B.; investigation, A.S.; resources, A.B.E. and C.T.T.; data curation, E.M.B. and S.U.; writing – original draft, A.S.; writing – review and editing, A.S. and C.T.T.; visualization, A.S.; supervision, C.T.T.; project administration, C.T.T.; funding acquisition, C.T.T.

## Declaration of interests

The authors declare no competing interests.

## Declaration of generative AI and AI-assisted technologies in the writing process

During the preparation of this work, the authors used Claude (versions 4–4.7; Anthropic) for language editing and drafting of selected manuscript sections. After using this tool, the authors reviewed and edited the content as needed and take full responsibility for the content of the publication. All scientific content, data analysis, interpretation, and conclusions are the sole responsibility of the authors.

## STAR★Methods

### Key resources table

**Table:** 

REAGENT or RESOURCE	SOURCE	IDENTIFIER
**Antibodies**		

Mouse anti-LAMA5	Atlas Antibodies	Cat# AMAb91124; RRID:AB_2665809; 1:1000 (WB)
Rabbit anti-beta-actin	Abcam	Cat# ab8227; RRID:AB_2305186; 1:1000 (WB)
Goat anti-mouse IgG, HRP	Invitrogen	Cat# 31430; RRID:AB_228307; 1:10000 (WB)
Goat anti-rabbit IgG, HRP	Cell Signaling Technology	Cat# 7074; RRID:AB_2099233; 1:10000 (WB)
Mouse anti-aggrecan (ACAN)	Abcam	Cat# ab3778; RRID:AB_304071; 1:500 (IF)
Rabbit anti-LAMA5	Abcam	Cat# ab220399; discontinued (no RRID assigned); 1:500 (IF)
Alexa Fluor 488 goat anti-mouse IgG	Invitrogen	Cat# A-11001; RRID:AB_2534069; 1:500 (IF)
Alexa Fluor 594 goat anti-rabbit IgG	Invitrogen	Cat# A-11012; RRID:AB_2534079; 1:500 (IF)

**Biological samples**		

HUVEC-conditioned medium (passage 0, freshly isolated from umbilical cords)	AG Cicha, University Hospital Erlangen (this study)	N/A

**Chemicals, peptides, and recombinant proteins**		

DMEM, high glucose	Gibco	Cat# 11965092
DMEM, low glucose	Gibco	Cat# 11885084
Keratinocyte Serum-Free Medium (KSFM)	Gibco	Cat# 17005042
alpha-MEM Eagle Medium	PAN Biotech	Cat# P04-21500
Fetal bovine serum (FBS)	Sigma-Aldrich/Roth	Cat# F7524-500 ML/286K.1
Opti-MEM I Reduced Serum Medium	Gibco	Cat# 31985070
Penicillin-Streptomycin	Gibco	Cat# 15140122
Endothelial Cell Growth Medium (ready-to-use)	PromoCell	Cat# C-22010
Y-27632 ROCK inhibitor	BD Biosciences	Cat# 563802
Recombinant human Laminin-521	Gibco	Cat# A29249
Recombinant human FGF-2	PeproTech/Gibco	Cat# 17823303
Thermostable recombinant human FGF-2	Sigma-Aldrich	Cat# GF446M
TrueCut Cas9 Protein v2	Invitrogen	Cat# A36499
Dexamethasone	Sigma-Aldrich	Cat# D4902-25 MG
L-ascorbic acid 2-phosphate sesquimagnesium salt hydrate	Sigma-Aldrich	Cat# COM448655215-5G
Disodium beta-glycerophosphate pentahydrate	Sigma-Aldrich	Cat# 35675-50 GM
SuperSignal Western Blot Enhancer	Thermo Scientific	Cat# 37035
Alcian blue 8GX	Sigma-Aldrich	Cat# A5268
Alizarin Red Solution	Merck Millipore	Cat# TMS-008-C
DAPI	Thermo Fisher Scientific	Cat# D1306; 1:1000
Aqua Polymount	Polysciences	Cat# 18606

**Critical commercial assays**		

StemPro Chondrogenesis Differentiation Kit	Gibco	Cat# A1007101
Lipofectamine CRISPRMAX	Invitrogen	Cat# CMAX00015
Platinum Direct PCR Universal Master Mix	Invitrogen	Cat# A44647100
BigDye Terminator v3.1 Cycle Sequencing Kit	Applied Biosystems	Cat# 4337455
SuperSignal West Femto Maximum Sensitivity Substrate	Thermo Scientific	Cat# 34095
TaqMan Gene Expression Assay: COL10A1	Applied Biosystems	Cat# Hs00166657_m1
TaqMan Gene Expression Assay: WNT7A	Applied Biosystems	Cat# Hs01114990_m1
TaqMan Gene Expression Assay: FLI1	Applied Biosystems	Cat# Hs00956709_m1
TaqMan Gene Expression Assay: SPP1	Applied Biosystems	Cat# Hs00959010_m1
TaqMan Gene Expression Assay: CDH1	Applied Biosystems	Cat# Hs01023895_m1
TaqMan Gene Expression Assay: RPLP0 (housekeeping)	Applied Biosystems	Cat# 4310879E

**Deposited data**		

Bulk RNA-seq raw data	ArrayExpress	E-MTAB-16566
Source data (Western blots, brightfield spheroids, immunofluorescence, RT-qPCR)	Mendeley Data	https://doi.org/10.17632/x92s3b23cz.1
Human fetal hindlimb single-cell atlas (re-used)	ArrayExpress	E-MTAB-8813
Mouse forelimb single-cell atlas (re-used)	Gene Expression Omnibus	GSE185940

**Experimental models: cell Lines**		

Human urine-derived stem cells (USCs); single male donor	Schulz et al. (companion study); see STAR Methods	Human primary cells
LAMA5 knockout USC clones (three independent CRISPR-Cas9-edited clones)	This paper	N/A

**Oligonucleotides**		

crRNA targeting LAMA5: TCCCTGGTGAACGGACGTCC	Integrated DNA Technologies	N/A
Alt-R CRISPR-Cas9 tracrRNA, ATTO 550	Integrated DNA Technologies	Cat# 1075927
F1 primer (CRISPR target): GAGAACGGAGAGGTGGGTAG	Integrated DNA Technologies	N/A
F2 primer (CRISPR target): ATCTGCACCACCGAGTACTC	Integrated DNA Technologies	N/A
R1 primer (CRISPR target): GCCTCCGATGCTGATATCCT	Integrated DNA Technologies	N/A
R2 primer (CRISPR target): GTGTTGGTACGCAGGAAGC	Integrated DNA Technologies	N/A

**Software and algorithms**		

Original code (custom R analysis scripts and FIJI macros)	This paper; GitHub: https://github.com/alexschulzcell/LAMA5-paper	Zenodo: https://doi.org/10.5281/zenodo.20697901
R	R Foundation	v4.5.1; RRID:SCR_001905
FIJI/ImageJ	Schindelin et al.	RRID:SCR_002285
DESeq2	Bioconductor	v1.48.1; RRID:SCR_015687
WGCNA	CRAN	v1.73.0; RRID:SCR_003302
ReactomePA	Bioconductor	v1.52.0; RRID:SCR_019316
enrichplot	Bioconductor	v1.28.4; RRID:SCR_026996
biomaRt	Bioconductor	v2.64.0; RRID:SCR_019214
STRINGdb	Bioconductor	v2.20.0; RRID:SCR_005223
igraph	CRAN	v2.1.4; RRID:SCR_019225
ggraph	CRAN	v2.2.1; RRID:SCR_021239
ggplot2	CRAN	v3.5.2; RRID:SCR_014601
ggrepel	CRAN	v0.9.6
pheatmap	CRAN	v1.0.13; RRID:SCR_016418
RColorBrewer	CRAN	v1.1.3; RRID:SCR_016697
clusterProfiler	Bioconductor	v4.16.0; RRID:SCR_016884
ComplexHeatmap	Bioconductor	v2.24.1; RRID:SCR_017270
circlize	CRAN	v0.4.16; RRID:SCR_002141
Seurat	Satija Lab/CRAN	v4; RRID:SCR_016341
SeuratDisk	GitHub (Satija Lab)	v0.0.0.9021; https://github.com/mojaveazure/seurat-disk
ColabFold/AlphaFold2	Mirdita et al./Jumper et al.	RRID:SCR_025453
PyMOL	Schrodinger, LLC	RRID:SCR_000305
GraphPad Prism	GraphPad Software	v10; RRID:SCR_002798
Sciugo (Western blot and gel composite generation)	Sciugo	https://sciugo.com

**Other**		

Mini-PROTEAN TGX Gel (4–15%)	Bio-Rad	Cat# 4561084
Trans-Blot Turbo Transfer System	Bio-Rad	Cat# 1704150
HiMark Pre-stained Protein Standard	Invitrogen	Cat# LC5699
Nunclon Sphera Ultra-Low Attachment Plates	Thermo Fisher Scientific	Cat# 174925
Neubauer-improved C-Chip disposable hemocytometer	NanoEnTek	Cat# DHC-N01

### Experimental model and study participant details

#### Human urine-derived stem cells

Human urine-derived stem cells (USCs) were isolated and expanded as described previously.^27^ Cells were cultured in a 1:1 mixture of DMEM high glucose and keratinocyte serum-free medium supplemented according to the manufacturer instructions and maintained under standard humidified conditions at 37 C and 5% CO2. Early-passage cultures were used for genome editing and differentiation experiments. All procedures involving human material were approved by the Ethics Committee of the Friedrich-Alexander-University Erlangen-Nurnberg (ref. 180_15 Bc). The USCs were derived from a single male donor. Because cells from only one individual were studied, the influence of sex or gender on the reported phenotypes could not be assessed, which represents a limitation of this study. The cell line was not formally authenticated by short tandem repeat (STR) profiling; however, the identity and stem-cell character of the parental USC line were independently characterized by single-cell RNA-sequencing in a companion study.^27^ The cells were not tested for mycoplasma contamination. For all experiments, three independent CRISPR-Cas9-edited LAMA5 knockout clones were compared with the parental wild-type line, with at least three biological replicates per condition as specified for each assay.

#### Clinical case ascertainment

Clinical data shown for the individual carrying *PITX1* p.M205L were de-identified and obtained in the context of clinical genetic diagnostics. Trio exome sequencing was followed by Sanger confirmation of the variant, and the summarized phenotypic findings are provided in Table S1 and Figure S5C of the Supplemental Information.

### Method details

#### Generation and validation of *LAMA5* knockout clones

CRISPR-Cas9 editing of *LAMA5* was performed using the Alt-R system with predesigned crRNA, tracrRNA, and recombinant Cas9 according to the manufacturer instructions. Ribonucleoprotein complexes were introduced into USCs with Lipofectamine CRISPRMAX, and ATTO550-positive cells were single-cell sorted by flow cytometry. Clones were expanded transiently on recombinant laminin-521 in the presence of Y-27632, screened by PCR across the targeted locus, and verified by Sanger sequencing. Three independent frameshift knockout clones were selected for downstream analyses. Loss of *LAMA5* transcript and protein was confirmed by RT-qPCR and western blotting.

#### Differentiation assays and staining

USCs were differentiated toward osteogenic or chondrogenic lineages using previously described conditions and commercial induction media where indicated. The parental USC line used for CRISPR editing was the same as that characterized in the single-cell study by Schulz et al.^27^; wild-type Alcian blue and Alizarin red staining panels shown in Figure 1B are therefore shared with that companion study. Alizarin red and Alcian blue staining were used to assess mineralization and glycosaminoglycan-rich matrix, respectively. For transcriptomic comparisons, cells were harvested at matched differentiation time points across WT and knockout cultures.

#### Spheroid morphogenesis assay

For three-dimensional chondrogenic spheroids, 20,000 cells were seeded per well in ultra-low attachment 96-well plates, centrifuged to aggregate the cells, and cultured in chondrogenic medium for up to 3 weeks. Spheroids were imaged longitudinally by brightfield microscopy. For morphometric analysis, images were processed using a custom FIJI^28^ macro: images were thresholded (range 95–255) after contrast adjustment, converted to binary masks, despeckled, and spheroid area was quantified by particle analysis (size threshold 5,000–∞ μm^2^, circularity 0.1–1.0). Spheroid radius was inferred from the measured area assuming a circular cross-section (r = √(A/π)), and volume was estimated as a sphere (V = 4/3 · π · r^3^). Circularity was extracted directly from the particle analysis output. A pixel size of 0.37 μm was applied for scale calibration. Measurements were performed from at least three independent biological replicates.

Terminal samples were fixed and immunostained for aggrecan (ACAN) and LAMA5. Immunofluorescence images were acquired as z-stacks of 25 optical sections on a Zeiss Axio Observer.Z1 with ApoTome.2 optical sectioning at 20× magnification, using fixed exposure times applied consistently across all samples (DAPI: 10 ms, *ACAN*: 35 ms, *LAMA5*: 30 ms; three wildtype and four knockout lines). Images were processed using a custom FIJI macro: maximum intensity projections were generated independently for each channel, and images were normalized based on DAPI signal intensity by Otsu-thresholding the DAPI channel, measuring the mean intensity within the segmented nuclear area, and applying a uniform scaling factor relative to a reference image to all three channels to preserve relative signal ratios. Normalized images were converted to 8-bit with fixed display ranges applied consistently across all samples. Composite RGB images were generated by merging all channels in FIJI.^28^

#### RNA extraction, RT-qPCR, and western blotting

RNA was isolated by column-based kits with genomic DNA removal. cDNA synthesis was performed with LunaScript RT SuperMix. RT-qPCR used TaqMan assays for *SPP1*, *COL10A1*, *WNT7A*, *FLI1* and *CDH1* with at least three biological replicates and normalization to the endogenous control included in the assay workflow. Western blots were performed on 4%–15% gradient gels with anti-*LAMA5* and anti-beta-actin antibodies, followed by HRP-conjugated secondary antibodies and chemiluminescent detection. Unedited blot images are provided in Figure S1B. Composite Western blot and PCR gel figure panels (Figures 1A, 1C, and S1B) were assembled and annotated using Sciugo (https://sciugo.com).

#### Bulk RNA sequencing and differential expression

Bulk RNA-seq libraries were prepared as described previously.^27^ Differential expression analysis was performed with DESeq2 (v1.48.1). Genes with adjusted *p* value <0.05 were considered differentially expressed. Variance-stabilized data were used for principal component analysis, heatmaps, and downstream network analyses. Sequencing quality control and principal component analysis are provided in Figure S1C.

#### Pathway enrichment and network analysis

Reactome enrichment analysis was performed on differentially expressed genes filtered at adjusted *p* value <0.05 and |log2 fold change| > 1 after identifier conversion with biomaRt. STRING-based protein interaction visualization used a minimum combined confidence score of 0.7 and was restricted to the largest connected component for display. Weighted gene co-expression network analysis (WGCNA) was performed on filtered expression matrices using unsigned topology, soft-thresholding power 9, minimum module size 30, and mergeCutHeight 0.25. Additional analytical details and the scale-free topology assessment are provided in the Supplemental Information and Figure S4A.

#### Single-cell atlas mapping, GWAS context, and structural modeling

Gene-expression patterns were projected onto a human fetal hindlimb single-cell atlas (E-MTAB-8813; 9 donors) and a mouse forelimb atlas (GSE185940) analyzed in Seurat. GWAS contextualization used summary statistics from a large meta-analysis of adult height, visualized in gene-centered windows. Structural models for wild-type *PITX1* and p.M205L were generated with ColabFold/AlphaFold2 and inspected in PyMOL. Extended computational details are provided in the supplemental information.^14^^,^^15^^,^^29^

#### LiCl rescue experiments

WT and KO cells were cultured on uncoated or laminin-521-coated plates and WNT signaling was activated by treatment with 20 mM lithium chloride (LiCl) for 24 h prior to harvest. *FLI1* expression was measured by RT-qPCR. KO2 cells with or without LiCl were additionally analyzed by RNA-seq. Rescue indices were calculated from batch-corrected, variance-stabilized expression values as *rescue index = (mean expression in KO + LiCl − mean expression in KO)/(mean expression in WT − mean expression in KO)*, such that a value of 0 corresponds to no rescue, 1 corresponds to full restoration to WT levels, and negative values indicate further deviation from WT in the direction opposite to rescue. Rescue indices were computed separately for the genes contributing to the embryonic limb morphogenesis GO term enrichment of the lightgreen module (Figure 5D) and for the top 15 lightgreen hub genes (Figure S5B).

Transcripts-per-million (TPM) values for canonical WNT/β-catenin target genes (*AXIN2*, *LEF1*, *TCF7*, *NKD1*, *SP5*, *CCND1*) were extracted from the variance-stabilized expression matrix and visualized as bar plots with overlaid individual replicate values for WT vs. KO and KO vs. KO + LiCl comparisons; significance was annotated from DESeq2 adjusted *p* values (Figure S3).

#### Proliferation assay

WT and *LAMA5* KO2 cells were seeded at 500 cells per well into 96-well plates that had been either left uncoated or pre-coated with recombinant laminin-521 (BioLamina), as described above. Cells were maintained under standard expansion conditions and harvested on consecutive days from day 1 to day 7 by detachment with 50 μL of trypsin and resuspension in culture medium. Live cell counts were obtained by manual counting on Neubauer-improved C-Chip disposable hemocytometers (NanoEnTek) without trypan blue exclusion. Three wells were seeded per condition from a single cell preparation; counts therefore represent technical replicates from one biological experiment. Growth curves were fitted with a logistic model and visualized in GraphPad Prism (Figure S1D).

#### HUVEC-conditioned medium

Conditioned medium was kindly provided by the laboratory of Prof. Iwona Cicha (Cardiovascular Nanomedicine Unit, Section of Experimental Oncology and Nanomedicine, Department of Otorhinolaryngology, Head and Neck Surgery, University Hospital Erlangen). Human umbilical vein endothelial cells (HUVECs) were freshly isolated in-house from umbilical cords obtained with institutional ethics approval (21-331-B, 06.10.2021) and cultured at passage 0 in Endothelial Cell Growth Medium Ready-to-use (PromoCell, C-22010). HUVEC-conditioned medium was collected from confluent passage-0 cultures, sterile-filtered, aliquoted, and stored at −80 °C until use.

Endothelial-input responsiveness of *LAMA5*-KO chondrogenic cultures was assessed by supplementing 2D chondrogenic induction medium (StemPro Chondrogenesis Kit) with 20% HUVEC-CM (final concentration in induction medium) for *LAMA5*-KO2 USCs. Medium was refreshed every 48 h. Two consecutive 48-h CM-treatment cycles were applied, and cells were harvested for RT-qPCR at day 5 of chondrogenic induction, corresponding to approximately 96 h of cumulative CM exposure with the final CM refresh 48 h before harvest. Parallel KO2 wells were maintained in chondrogenic medium without HUVEC-CM as the within-genotype control. The spheroid (3D) format was not used for this readout; all Figure 6 samples were processed in 2D culture.

RT-qPCR was performed for *WNT7A*, *FLI1*, and *CDH1* using TaqMan assays (key resources table), with normalization to the *RPLP0* housekeeping transcript. *n* = 4 independent differentiation runs (independent cell preparations and induction events of KO2) per condition. Threshold cycle (Ct) values were processed using the same pipeline as the other RT-qPCR experiments in this study: per-sample × per-target outlier removal at 1.5 × interquartile range (IQR); ΔCt = mean(target Ct) − mean(housekeeping Ct); ΔΔCt referenced to the mean ΔCt of the untreated KO2 group; Relative quantificantion (RQ) = 2ˆ(−ΔΔCt). Statistical comparison between HUVEC-CM and untreated groups was performed by unpaired Welch’s two-sample *t* test on ΔΔCt values per gene. Owing to limited availability of HUVEC-conditioning material from the external source, the experiment was performed as a within-genotype comparison without a matched basal-medium mock control. Effects are therefore interpreted as hypothesis-supporting rather than mechanistically definitive.

### Quantification and statistical analysis

#### Statistical analysis

No statistical methods were used to predetermine sample size. Throughout this study, biological replicates were chosen to match the question being asked. Transcriptome-wide analyses, RT-qPCR validation of differentially expressed genes, and morphological characterization of chondrogenic spheroids were performed across all three independently derived KO clones (KO1, KO2, KO3), with each clone counted as one biological replicate. For spheroid imaging and morphometry, multiple aggregates per clone and time point were pooled to increase statistical power for the architectural readouts, while the underlying biological replication remained at the level of independent clones. In contrast, experiments addressing functional perturbation or candidate niche responsiveness—the LiCl rescue, the proliferation assay, and the HUVEC-conditioned medium rescue—were carried out in a single KO clone (KO2) to enable repeated independent runs of the same genetic background; in these cases, biological replicates refer to independent differentiation runs (independent cell preparations and induction events) of that clone, unless explicitly noted otherwise. The proliferation assay represents an explicitly stated exception: three wells from a single cell preparation, reported as technical replicates within one biological experiment, as detailed in the corresponding methods subsection. RT-qPCR data were analyzed using unpaired Welch t tests on DeltaDeltaCt values. Spheroid morphometry was analyzed using mixed-effects models or repeated-measures testing as appropriate for the experimental design, as specified in the corresponding figure legends. Adjusted *p* values for RNA-seq were calculated with the Benjamini-Hochberg procedure implemented in DESeq2. Statistical details for each experiment are reported in the figure legends.
