# Supplementary material for: LAMA5 links extracellular matrix organization to a candidate WNT-associated endothelial signaling niche during human chondrogenesis
Source: iScience. 2026 Jul 10;29(8):116687. doi: 10.1016/j.isci.2026.116687 (PMC13380422; doi:10.1016/j.isci.2026.116687)
Supplement: Document S1. Figures S1–S5 and Table S1 and supplemental methods [file mmc1.pdf]

iScience, Volume *29*

Supplemental information

LAMA5 links extracellular matrix organization to a candidate WNT-associated endothelial signaling niche during human chondrogenesis

Alexander Schulz, Emily M. Brockmann, Steffen Uebe, Arif B. Ekici, and Christian T. Thiel

Supplemental Information

Supplemental Methods

Extended analytical details supporting the main manuscript are provided below.

Spheroid image processing

Brightfield images for morphometric analysis were processed in FIJI using a custom macro. Images were contrast-adjusted (display range 80–100), thresholded (95–255, black background), converted to binary masks, and despeckled. Spheroid boundaries were detected by particle analysis with a minimum area of 5,000 μm^2 and circularity range 0.1–1.0. Pixel size was calibrated to 0.37 µm/pixel. Spheroid radius was derived from the measured cross-sectional area as $r = \sqrt{(A/\pi)}$, and volume was estimated assuming spherical geometry as $V = 4/3 \cdot \pi \cdot r^3$. Circularity was recorded directly from the particle analysis output.

Immunofluorescence z-stack images were processed using a separate custom FIJI macro. Maximum intensity projections (max intensity) were computed across 25 z-planes for each channel independently. To enable quantitative comparison across biological samples, a DAPI-based normalization was applied: nuclei were segmented by Otsu thresholding on the DAPI projection, the mean pixel intensity within the segmented nuclear area was measured using a binary selection mask, and a per-image scaling factor was calculated relative to a fixed reference value. This factor was applied uniformly to all three channels (DAPI, ACAN, LAMA5) to preserve relative signal ratios between channels. Scaling factors were capped between 0.2 and 5.0 to prevent overcorrection of outlier images. Normalized 16-bit images were converted to 8-bit using fixed display ranges (DAPI: 0–20, green channel: 0–12, red channel: 0–12, in normalized arbitrary units) applied identically across all samples. Both macros are available upon request.

RNA-seq library preparation and sequencing

RNA was extracted from USC samples using the RNeasy Mini Kit (Qiagen) with on-column DNase digestion according to the manufacturer's protocol. cDNA libraries were prepared using the Illumina Stranded mRNA Kit. Paired-end sequencing (fragment length 159 bp) was performed on an Illumina NovaSeq 6000 platform. Raw data were converted to reads and demultiplexed using Illumina DRAGEN Software (v3.8.4). Ribosomal and other unwanted RNA reads were removed using BWA-MEM (v0.7.17) in combination with SAMtools (v1.17), and reads were converted back to FASTQ format using SamToFastq (GATK v4.2.1.0). Per-lane reads were aligned to the hg38 reference genome with Ensembl gene annotations (release 110) using STAR (v2.7.10a). Per-sample alignments were merged using MergeSamFiles (Picard v2.25.4). A count matrix was generated using featureCounts (v2.0.1) with the same Ensembl annotation. Alignment and quantification metrics were used for quality assessment.

Reactome pathway enrichment analysis

Differentially expressed genes identified by DESeq2 were filtered using adjusted p value < 0.05 and absolute log2 fold change > 1. Ensembl gene identifiers were mapped to Entrez Gene IDs using biomaRt, and Reactome pathway enrichment was performed with enrichPathway in ReactomePA with Homo sapiens as organism. Pathways with nominal $p < 0.05$ were considered for descriptive reporting in the supplemental analyses.

STRING protein-protein interaction analysis

Genes associated with the Reactome pathway Cell junction organization were intersected with differentially expressed genes. High-confidence protein-protein interactions were retrieved using STRINGdb with a minimum combined confidence score of 0.7. Networks were visualized in igraph/ggraph after filtering to the largest connected component and retaining genes with absolute log2 fold change >= 1.

Weighted gene co-expression network analysis

Raw count matrices were filtered to retain genes with counts > 1 in at least four samples, transformed with the DESeq2 variance-stabilizing workflow, and analyzed using WGCNA. Soft-thresholding power 9 was selected using scale-free topology criteria, and blockwiseModules was run with unsigned topology, minimum module size 30, and mergeCutHeight 0.25. Hub genes were defined from module membership rankings.

PITX1 structural modeling

Wild-type and p.M205L *PITX1* models were generated using ColabFold/AlphaFold2 default settings and visualized in PyMOL.

Single-cell RNA-seq analysis – human fetal growth plate

The annotated h5ad object (221114LimbCellranger3annotated.minimal.h5ad) for project E-MTAB-8813 was downloaded from the Human Cell Atlas Data Portal (contributor-generated matrices). The object was loaded using the anndata R package, converted to a Seurat object, and filtered to hindlimb samples (tissue = "LowerLimb"), yielding 93,918 cells from 9 biological donors spanning 5.1–9.3 post-conception weeks. Data were normalized using NormalizeData, variable features identified (nfeatures = 3,000), scaled, and dimensionality-reduced by PCA (30 PCs). Graph-based clustering (FindNeighbors/FindClusters, resolution = 0.5) and UMAP projection were performed on the top 30 PCs. Cell types were annotated by computing module scores (AddModuleScore) for chondrocyte (SOX9, COL2A1, ACAN), perichondrial (PRRX1, COL1A1, TWIST1), and endothelial (PECAM1, KDR, EMCN) marker sets; each cluster was assigned the identity with the highest mean module score.

To visualize the spatial distribution of key network genes within the UMAP embedding, cells were annotated based on normalized expression of *LAMA5*, *WNT7A*, *TFAP2A*, *GRHL2*, *PITX1*, and *FLI1*. Co-expressing cell populations were defined in priority order: $LAMA5^{+}$/$WNT7A^{+}$ double-positive, *LAMA5*$^{+}$ single-positive, *WNT7A*$^{+}$ single-positive, *TFAP2A*$^{+}$ and/or *GRHL2*$^{+}$ positive, *PITX1*$^{+}$/*FLI1*$^{+}$ double-positive, and $PITX1^{+}$ or $FLI1^{+}$ single-positive. Remaining cells were colored by coarse cell-type identity. Any normalized expression > 0 was considered positive.

Single-cell RNA-seq analysis – mouse limb

Raw count matrices for 103 sequencing libraries (GSE185940) representing multiple biological replicates across five embryonic stages (E10.5–E14.5) were extracted from the GSE185940_RAW.tar archive, loaded, prefixed with GSM identifiers to ensure unique cell barcodes, and concatenated. A Seurat object was created (min.cells = 3, min.features = 200) and processed identically to the human dataset (NormalizeData, FindVariableFeatures nfeatures = 2,000, ScaleData, PCA 30 PCs, FindNeighbors/FindClusters resolution = 0.6, UMAP dims 1:20). Cell metadata were matched to the provided metadata file via cell barcode. Cell-type annotation and gene expression followed the same

strategy as described for the human dataset, using mouse gene symbols (*Lama5*, *Wnt7a*, *Tfap2a*, *Grhl2*, *Pitx1*, *Fli1*).

Height GWAS visualization

Height GWAS summary statistics from Yengo et al.[S1] were processed in R using data.table and ggplot2. Chromosomal positions were converted to cumulative base-pair coordinates by adding per-chromosome offsets based on maximum observed positions. For visualization, background SNPs were pruned to one lead SNP per 1 Mb window per chromosome (highest $-\log_{10}(P)$ retained); all SNPs within ±250 kb of the gene bodies of *LAMA5*, *WNT7A*, *PITX1*, *FLI1*, *GRHL2*, and *TFAP2A* were retained at full resolution and colored by gene. $-\log_{10}(P)$ values were capped at 60 for display. Gene labels were placed at the top-scoring SNP per locus using ggrepel. The genome-wide significance threshold ($P < 5\times10^{-8}$) is indicated by a dashed line.

Supplemental Figures

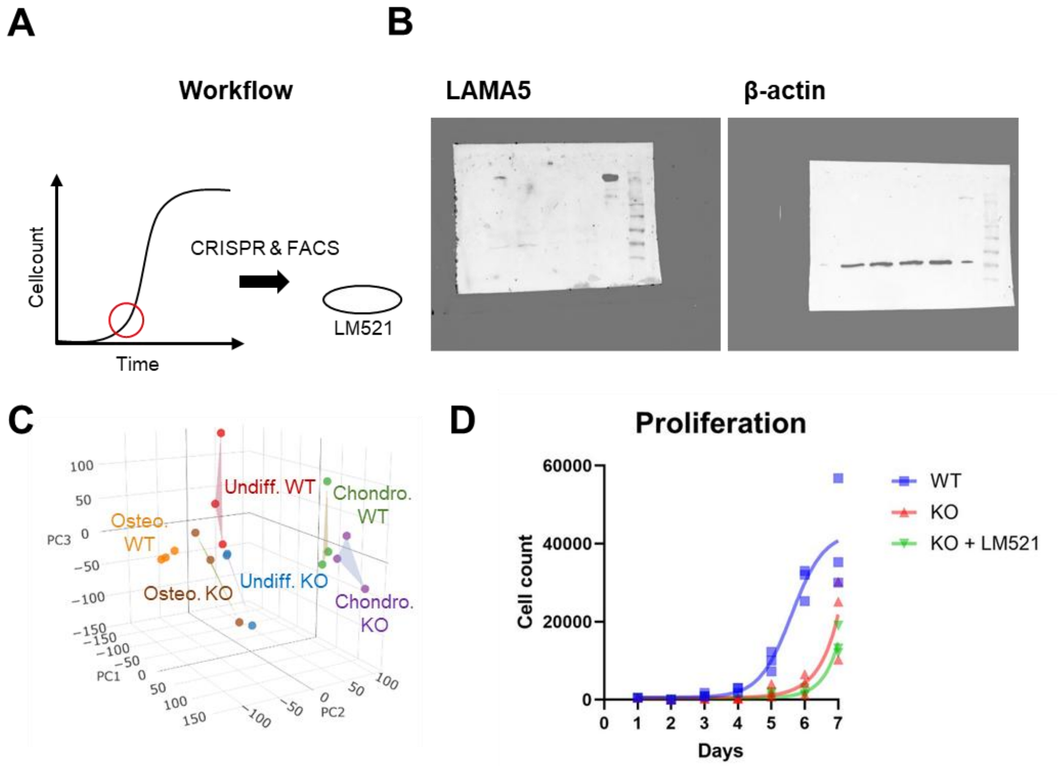

Figure S1. Cell model validation and knockout confirmation, Related to Figure 1.

(A) Schematic CRISPR workflow used for urine-derived stem cells. Transient coating with laminin-521 during clonal derivation supported the initial S-shaped growth curve characteristic of CRISPR-edited single-cell clones.

(B) Unedited western blot source data corresponding to the cropped blots in Figure 1A.

(C) Principal component analysis of bulk RNA-seq data. Sample labels indicate genotype and differentiation condition (Undiff., Osteo WT, Osteo KO, Chondro. WT, Chondro. KO).

(D) Proliferation assay. Live cell counts of WT (blue), *LAMA5* KO2 (red), and laminin-521-coated *LAMA5* KO2 (green) cells seeded at 500 cells/well in 96-well plates and harvested daily from day 1 to day 7. Each point represents one well count (n = 3 wells per condition per day from a single biological experiment); curves represent logistic fits. KO2 cells reached lower cell counts than WT cells by day 7, and pre-coating with recombinant laminin-521 did not restore proliferation toward WT levels.

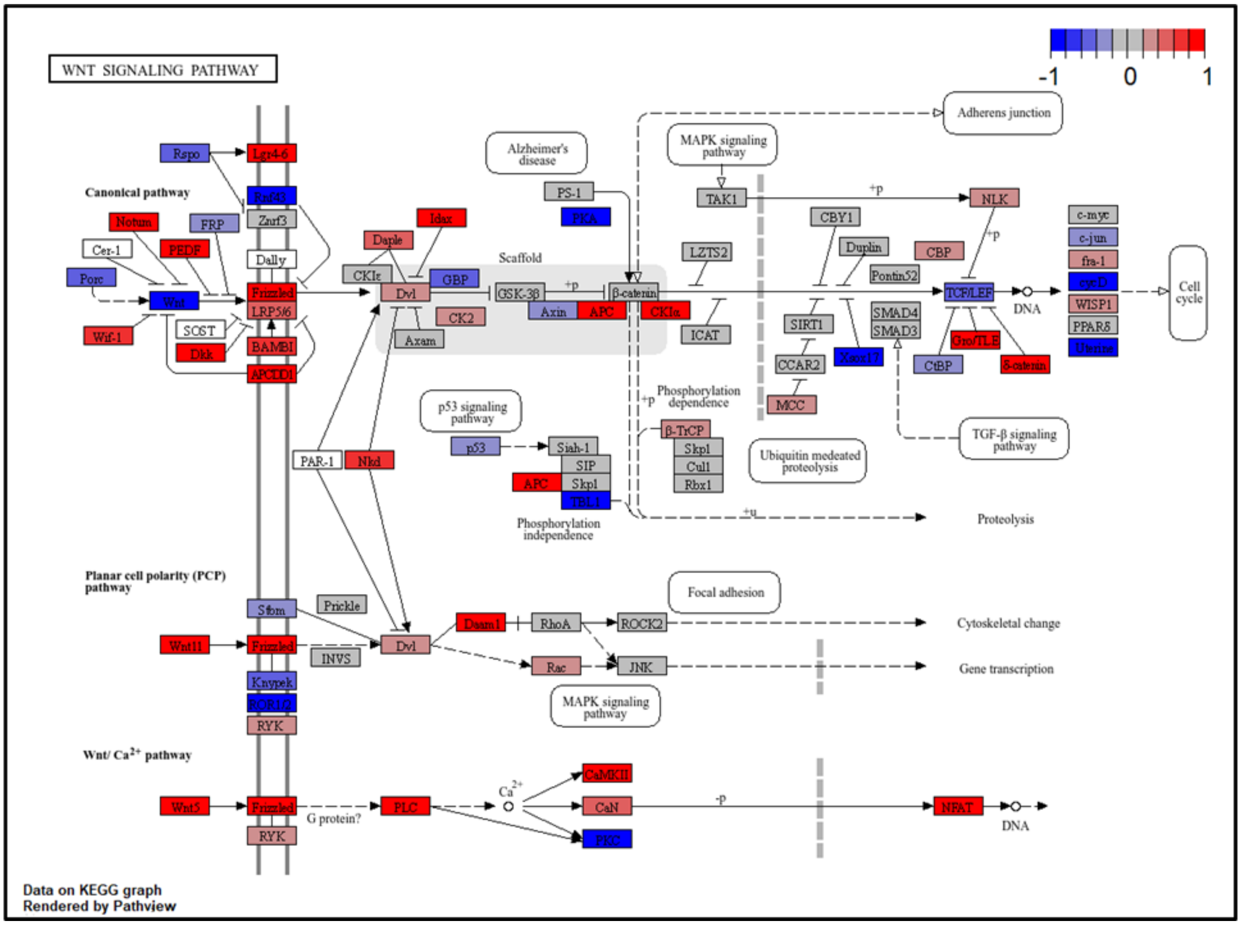

Figure S2. Extended WNT pathway analysis, Related to Figure 2.

KEGG pathway overlay (Pathview) of canonical WNT signaling components with differential expression data from KO versus WT chondrogenic cultures. Consistent with the WNT heatmap in Figure 2F, the pathway shows broad dysregulation rather than strictly unidirectional suppression.

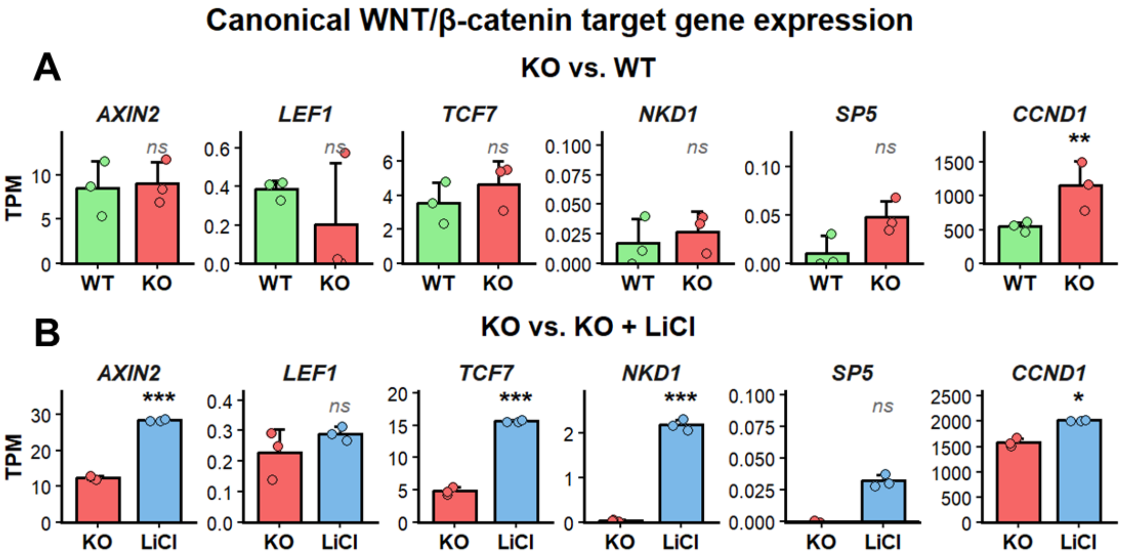

Figure S3. Transcript-level abundance of canonical β-catenin/TCF target genes in WT, KO, and LiCl-treated KO chondrogenic cultures, Related to Figures 2 and 5.

(A) Transcripts-per-million (TPM) values for canonical β-catenin/TCF target genes (AXIN2, LEF1, TCF7, NKD1, SP5, CCND1) in WT versus LAMA5 KO chondrogenic cultures, derived from variance-stabilized bulk RNA-seq data. Bars represent group means; overlaid points indicate individual biological replicates. With the exception of a modest but significant increase in CCND1, canonical target transcripts were not coordinately altered in KO cultures, consistent with a perturbation upstream of intracellular β-catenin activity rather than a canonical pathway suppression signature.

(B) TPM values for the same canonical target genes in KO2 cultures with and without 20 mM lithium chloride (LiCl, 24 h). LiCl strongly induced AXIN2, TCF7, NKD1, and CCND1, confirming that pharmacologic β-catenin stabilization effectively engages canonical TCF-driven transcription in this system. The partial and gene-specific rescue of the LAMA5-associated module by LiCl (Figure 5C; Figure S4A–B) therefore does not reflect a failure of canonical pathway activation.

Data are based on n = 3 biological replicates per condition. Significance is annotated from DESeq2 adjusted p values: * $p < 0.05$, ** $p < 0.01$, *** $p < 0.001$; ns, not significant.

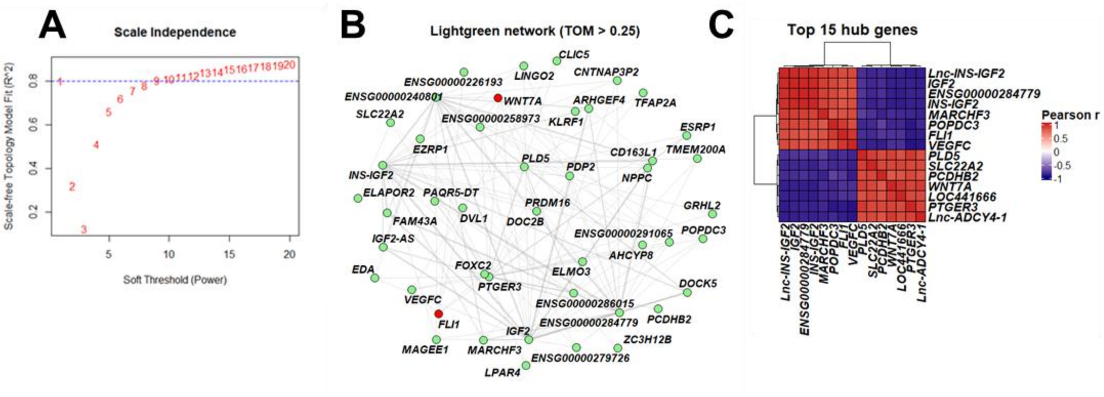

Figure S4. Extended WGCNA module characterization, Related to Figure 3.

(A) Scale-free topology analysis supporting WGCNA soft-thresholding power selection.

(B) Full network visualization of the lightgreen module (module membership > 0.25), showing dense intra-module connectivity among development-associated genes.

(C) Pairwise correlation heatmap of the top 15 hub genes within the lightgreen module.

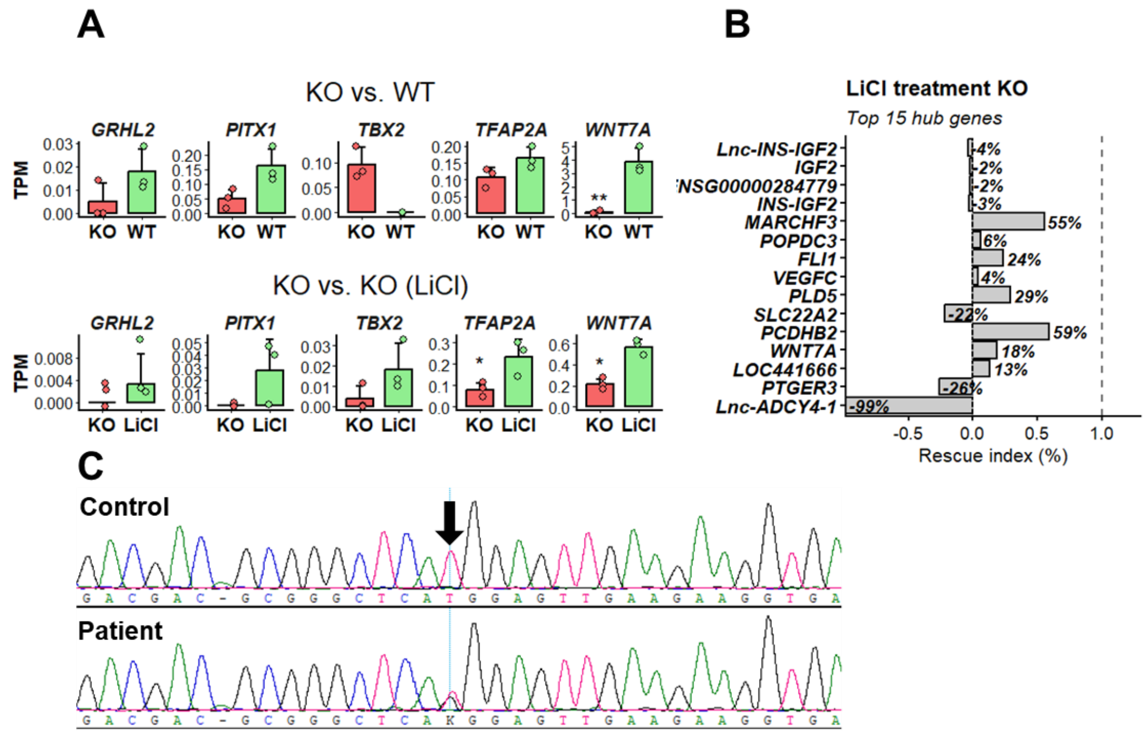

Figure S5. LiCl rescue details and clinical variant validation, Related to Figure 5.

(A) Transcripts-per-million (TPM) values for the embryonic-limb-morphogenesis (ELM) gene set (GO:0030326; WNT7A, PITX1, TFAP2A, GRHL2, TBX2) across WT, LAMA5-KO, and LiCl-treated KO conditions, derived from variance-stabilized bulk RNA-seq data. Bars represent group means; overlaid points indicate individual biological replicates. Adjusted P values from DESeq2 are shown above pairwise comparisons. These panels provide the underlying per-gene distributions for the rescue-index summary in Figure 5D.

(B) Rescue index after LiCl treatment of KO2 cells for the top 15 hub genes of the LAMA5-associated lightgreen module, ranked by module membership (kME). Rescue index = (KO2+LiCl − KO2) / (WT − KO2), calculated from batch-corrected variance-stabilized expression as in Figure 5D. 100 % (dashed line) indicates restoration to WT level; 0 % (dotted line) indicates no change relative to untreated KO2; negative values indicate further divergence from WT. This panel extends the focused embryonic-limb-morphogenesis analysis in Figure 5D to the broader set of high-membership module genes (including IGF2, FLI1, VEGFC, PLD5, PTGER3, and lncRNAs of the IGF2 locus) and illustrates that LiCl rescue is partial and gene-specific across the module rather than uniform.

(C) Sanger sequencing confirmation of the PITX1 p.M205L variant in the proband.

Supplemental Tables

Table S1. Clinical, anthropometric, and molecular genetic characteristics of the de-identified individual with isolated short stature carrying *PITX1* p.M205L, Related to Figure 5.

Field	Entry
Sex	F
Age at examination	12 years
Height	131.4 cm (-3.3 SDS; <1st centile)
Weight / BMI	36.2 kg / 18.3 kg/m²
Key phenotypic features	Mild brachydactyly; broad chest; mildly low-set ears; short/broad forefeet; otherwise normal psychomotor development
Birth history	38 weeks; birth weight 2685 g; APGAR 9/10/10
Karyotype / CNV	46,XX; no pathogenic CNV detected
Test method	Trio exome sequencing plus Sanger confirmation
Variant	*PITX1* NM_002653.3, c.A613C (p.M205L), heterozygous

Supplemental References

[S1] Yengo, L., Vedantam, S., Marouli, E., Sidorenko, J., Bartell, E., Sakaue, S., Graff, M., Eliasen, A.U., Jiang, Y., Raghavan, S., et al. (2022). A saturated map of common genetic variants associated with human height. Nature *610*, 704–712. 10.1038/s41586-022-05275-y.
